# Characterization of exceptionally thermostable single-stranded DNA-binding proteins from *Thermotoga maritima *and *Thermotoga neapolitana*

**DOI:** 10.1186/1471-2180-10-260

**Published:** 2010-10-15

**Authors:** Marcin Olszewski, Anna Grot, Marek Wojciechowski, Marta Nowak, Małgorzata Mickiewicz, Józef Kur

**Affiliations:** 1 Gdańsk University of Technology, Department of Microbiology, ul. Narutowicza 11/12, 80-233 Gdańsk, Poland; 2 Gdańsk University of Technology, Department of Pharmaceutical Technology and Biochemistry, ul. Narutowicza 11/12, 80-233 Gdańsk, Poland

## Abstract

**Background:**

In recent years, there has been an increasing interest in SSBs because they find numerous applications in diverse molecular biology and analytical methods.

**Results:**

We report the characterization of single-stranded DNA binding proteins (SSBs) from the thermophilic bacteria *Thermotoga maritima *(*Tma*SSB) and *Thermotoga neapolitana *(*Tne*SSB). They are the smallest known bacterial SSB proteins, consisting of 141 and 142 amino acid residues with a calculated molecular mass of 16.30 and 16.58 kDa, respectively. The similarity between amino acid sequences of these proteins is very high: 90% identity and 95% similarity. Surprisingly, both *Tma*SSB and *Tne*SSB possess a quite low sequence similarity to *Escherichia coli *SSB (36 and 35% identity, 55 and 56% similarity, respectively). They are functional as homotetramers containing one single-stranded DNA binding domain (OB-fold) in each monomer. Agarose mobility assays indicated that the ssDNA-binding site for both proteins is salt independent, and fluorescence spectroscopy resulted in a size of 68 ± 2 nucleotides. The half-lives of *Tma*SSB and *Tne*SSB were 10 h and 12 h at 100°C, respectively. When analysed by differential scanning microcalorimetry (DSC) the melting temperature (*T*_m_) was 109.3°C and 112.5°C for *Tma*SSB and *Tne*SSB, respectively.

**Conclusion:**

The results showed that *Tma*SSB and *Tne*SSB are the most thermostable SSB proteins identified to date, offering an attractive alternative to *Taq*SSB and *Tth*SSB in molecular biology applications, especially with using high temperature e. g. polymerase chain reaction (PCR).

## Background

Single-stranded DNA-binding (SSB) proteins play an essential role in all *in vivo *processes involving ssDNA. They interact with ssDNA and RNA, in an independent from sequence manner, preventing single-stranded nucleic acids from hybridization and degradation by nucleases [1]. SSB proteins play a central role in DNA replication, repair and recombination [2-4]. They have been identified in all classes of organisms, performing similar functions but displaying little sequence similarity and very different ssDNA binding properties. Based on their oligomeric state, SSBs can be classified into four groups: monomeric, homodimeric, heterotrimeric and homotetrameric. A prominent feature of all SSBs is that the DNA-binding domain is made up of a conserved motif, the OB (oligonucleotide binding) fold [5]. Most of the bacterial SSBs exist as homotetramers. However, recent discoveries have shown that SSB proteins from the genera *Thermus *and *Deinococcus *possess a different architecture. SSB proteins in these bacteria are homodimeric, with each SSB monomer encoding two OB folds linked by a conserved spacer sequence [6-9].

At present, with the exception of SSB from *Thermoanaerobacter tengcongensis *[11], all bacterial thermostable SSBs belong to the *Deinococcus-Thermus *phylum. They have been found in *T. aquaticus *[6,12], *T. thermophilus *[6,12], *D. radiodurans *[7], *D. geothermalis *[13], *D. murrayi *[14], *D. radiopugnans *[15], *D. grandis *and *D. proteolyticus *[16]. In addition, thermostable SSBs have also been found in thermophilic crenarchaea e. g. *Sulfolobus solfataricus *[17].

*Thermotoga maritima *and *T. neapolitana *are strictly anaerobic heterotrophic Eubacteria growing in marine environments at temperatures ranging from 50 to 95°C. Their DNA base composition is 46 and 41 mol% guanine+cytosine, respectively [18,19]. Among the Eubacteria sequenced to date, *T. maritima *has the highest percentage (24%) of genes that are highly similar to archeal genes. The observed conservation of gene order between *T. maritima *and Archaea in many of the clustered regions suggests that lateral gene transfer may have occurred between thermophilic Eubacteria and Archaea [20].

Genomes of bacteria presented in the NCBI database have been screened in search for *ssb *gene homologs and their organization. In all the genomes, one or more genes coding for an SSB homolog were found [21]. On the basis of the *ssb *gene organization and the number of *ssb *paralogs, they classified bacteria in four different groups. *T. maritima *was classified as group II, which contains bacteria with the *ssb *gene organization *rpsF-ssb-rpsR*.

In the present study the purification and characterization of two highly thermostable SSB proteins from *T. maritima *and *T. neapolitana *are described.

## Results

### Sequence analysis

The *Tma*SSB and *Tne*SSB proteins contained 141 and 142 amino acid residues with a calculated molecular mass of 16.30 and 16.58 kDa, respectively. They are the smallest prokaryotic SSB proteins so far identified (*E. coli *SSB with N-terminal methionine consists of 178 amino acid residues). Analysis of the primary structures by RPS-BLAST [22] revealed the presence of two distinctive regions: one putative OB-fold domain (from amino acid 1-120) and one C-terminal domain that contains five conserved DEPPF terminal amino acids, which are common in all known bacterial SSB proteins.

**Figure 1:**
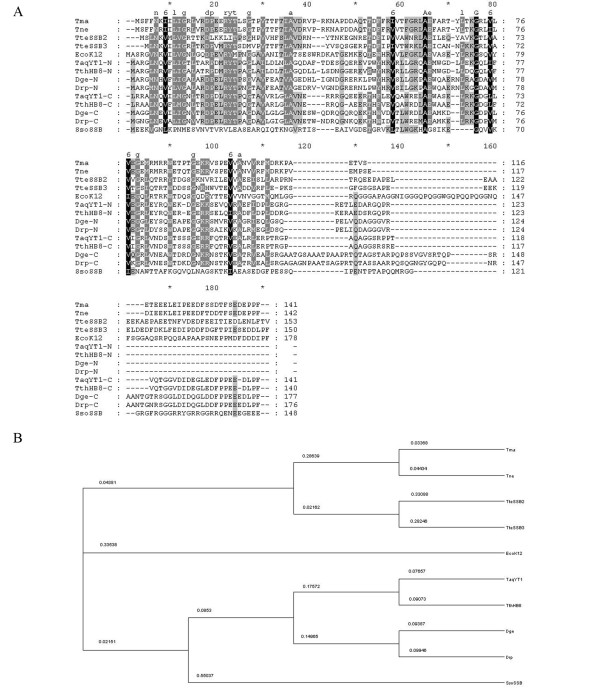
**A: Multiple amino acid sequence alignment of SSB proteins**. Alignment was performed by dividing amino acids into six similarity groups: group 1, V, L, I and M; group 2, W, F and Y; group 3, E and D; group 4, K and R; group 5, Q and D; group 6, S and T. White fonts on black boxes denote 100% identity; white fonts on grey boxes show <80% similarity; black fonts on grey boxes show <60% similarity. B: Dendogram of SSB proteins. Abbreviations: Tma, *T. maritima *strain MSB8; Tne, *T. neapolitana*; EcoK12, *E. coli *K12; TteSSB2, TteSSB3, *T. tengcongensis *strain MB4; Taq, *T. aquaticus *strain YT1; Tth, *T. thermophilus *strain HB8; Dge, *D. geothermalis; *Drp, *D. radiopugnans *strain R1; Sso, *S. solfataricus *P2; N, N-terminal ssDNA-binding domain; C, C-terminal ssDNA-binding domain.

Figure 1 shows an alignment of amino acid sequences of *T. maritima*, *T. neapolitana, Thermoanaerobacter tengcongensis*, *Sulfolobus solfataricus *and *E. coli *SSB proteins containing one OB-fold domain for monomer, and *T. aquaticus*, *T. thermophilus*, *D. geothermalis *and *D. radiopugnans *thermostable SSB proteins containing two OB-fold domains for monomer. The similarity between the amino acid sequences of *Thermotoga *SSBs is very high: 90% identity and 95% similarity. Surprisingly, both *Thermotoga *SSBs had a quite low sequence similarity to *Escherichia coli *SSB (*Tma*SSB has 36% identity and 55% similarity, *Tne*SSB has 35% identity and 56% similarity), whereas the similarity to *Thermoanaerobacter tengcongensis *SSB3 was higher (63 and 64% similarity; 40 and 42% identity for *Tma*SSB and *Tne*SSB, respectively).

### Expression and purification of the recombinant TmaSSB and TneSSB proteins

**Figure 2:**
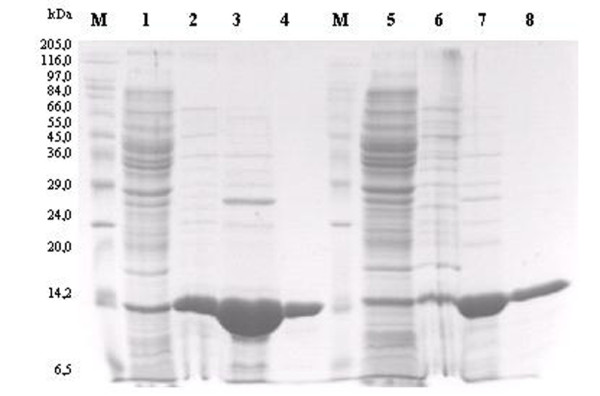
**Expression and purification of the *Tma*SSB and *Tne*SSB**. Proteins expression were obtained from the pET30Ek/LIC vector in BL(DE3)pLysS *E. coli *cells. Proteins were examined on 15% SDS-polyacrylamide gel. *Lane M*, Marker Wide Range (Sigma) with the molecular mass of proteins marked; *lanes 1 and 5*, soluble protein cell extracts after IPTG induction of protein expression (10 μl); *lanes 2 and 6*, *Tma*SSB and *Tne*SSB after heat treatment at 80°C for 20 min (10 μl); *lane 3 and 7*, *Tma*SSB and *Tne*SSB after chromatography on a QAE-cellulose column (10 μl); *lane 4 and 8*, *Tma*SSB and *Tne*SSB after chromatography on a ssDNA-cellulose column (10 μl).

Using the recombinant plasmid pETSSBTma or pETSSBTne, the expression of inducible proteins with the predicted size was excellent (Figure 2, lanes 1 and 5). Both proteins were expressed in a soluble form in the cytosol. Heat treatment resulted in considerably less contamination by the host proteins (Figure 2, lanes 2 and 6). The *E. coli *overexpression system used in this study produced about 40 and 35 mg of purified *Tma*SSB and *Tne*SSB protein, respectively, from 1 l of induced culture. The purity of the protein preparations was about 99% (Figure 2, lanes 4 and 8).

### Oligomerization status of the TmaSSB and TneSSB proteins

**Figure 3:**
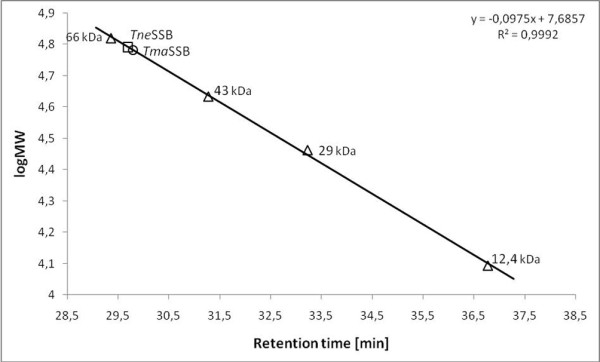
**Analytical gel filtration of *Tma*SSB and *Tne*SSB on Superdex HR 75 column**. A standard linear regression curve was generated by plotting the log of the molecular mass of the calibration proteins against their retention times (min) and is shown. The calibration proteins include bovine albumin (66 kDa), ovalbumin (43 kDa), carbon anhydrase (29 kDa) and cytochrome C (12.4 kDa).

Analysis of the purified proteins by SDS-PAGE revealed a single major band with a molecular mass of about 16 kDa for both proteins. In contrast, analysis by gel filtration chromatography revealed single peaks with a molecular mass of about 60.48 kDa for *Tma*SSB and 61.86 kDa for *Tne*SSB (Figure 3). This native molecular mass is approximately is 3.7 times the molecular mass of the monomer for both proteins. This confirmed our prediction that in solution the *Tma*SSB and *Tne*SSB proteins exist as homotetramers. Chemical cross-linking using glutaraldehyde confirmed the tetrameric state of the examined proteins (not shown).

### DNA-binding properties

**Figure 4:**
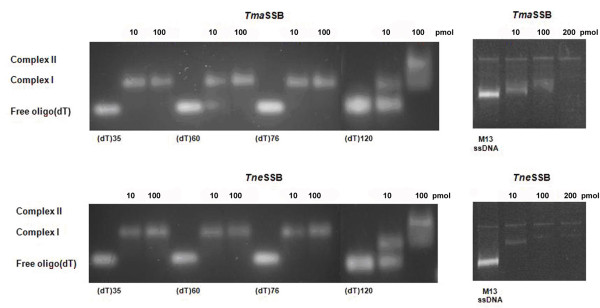
**Binding of *Tma*SSB and *Tne*SSB to oligo(dT) and to M13 ssDNA- gel mobility shift assays**.

When (dT)_35_, (dT)_60 _or (dT)_76 _were incubated with increasing amounts of *Tma*SSB or *Tne*SSB, a single band of reduced mobility was observed (Figure 4, complex I). Most of those oligonucleotides were shifted after addition of 10 pmol of SSBs, and the mobility of the shifted band remained constant at the higher protein amounts (100 pmol). One band of identical mobility was observed for (dT)_120 _at the low protein amounts, but a second band with a lower mobility appeared at the higher protein amounts (100 pmol; Figure 4, complex II)). These results suggest that *Tma*SSB and *Tne*SSB bind to (dT)_35_, (dT)_60 _or (dT)_76 _as one single homotetramer whereas two SSB homotetramers bind to (dT)_120_. Similar binding patterns were observed with the *Tma*SSB and *Tne*SSB proteins in different salt concentrations (2 or 100 mM NaCl).

The binding of the *Tma*SSB and *Tne*SSB proteins to the naturally occurring circular M13 ssDNA (6,407 nucleotides) was also examined. In this experiment, a fixed amount of M13 ssDNA was incubated with increasing amounts of SSB protein, and the resulting complexes were analyzed by agarose gel electrophoresis (Figure 4). When increasing amounts of *Tma*SSB or *Tne*SSB protein were added to M13 ssDNA, there was a progressive decrease in the mobility of the M13 ssDNA.

**Figure 5:**
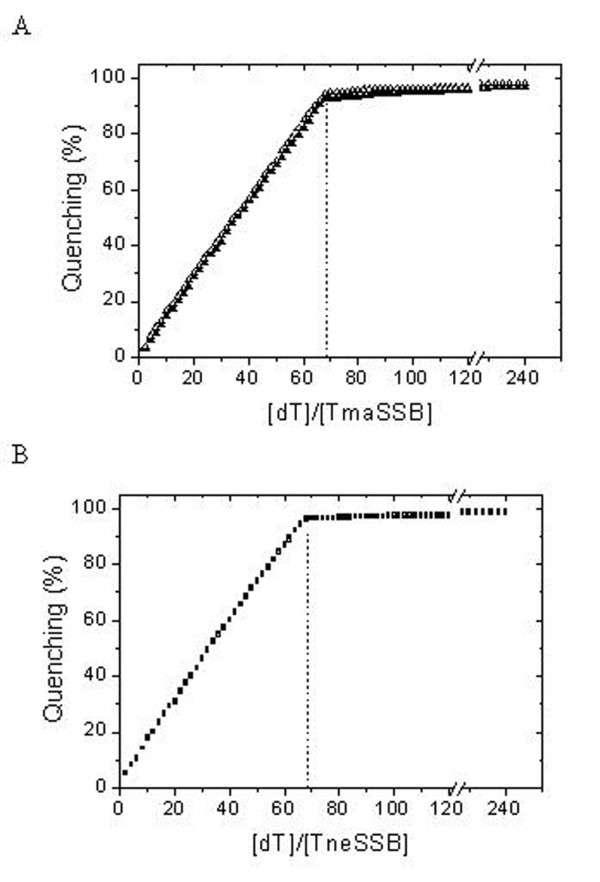
**Inverse fluorescence titration of *Tma*SSB and *Tne*SSB with (dT)_76_**. A 1 nM sample of *Tma*SSB (A) and *Tne*SSB (B) was titrated with (dT)_76 _at 2 mM NaCl (filled figures) or 100 mM NaCl (open figures) in binding buffer.

To further explore the binding properties of the examined SSB proteins, we used fluorescence spectroscopy. All bacterium SSB proteins (both homotetrameric and homodimeric) studied so far have shown a dramatic decrease of tryptophan fluorescence when binding to ssDNA. With an excitation wavelength of 295 nm, the emission spectrum of SSB proteins at 25°C had a maximum at 348 nm, which is consistent with tryptophan fluorescence. When adding a saturating quantity of ssDNA, the intrinsic fluorescence at 348 nm was quenched by 95% for both the *Tma*SSB and the *Tne*SSB proteins. The estimated size of the ssDNA binding site in the presence of 2 or 100 mM of NaCl for the *Tma*SSB and the *Tne*SSB proteins was 68 ± 2 nt (Figure 5). None binding-mode transition was observed when changing the ionic strength from low (2 mM NaCl) to high salt (100 mM NaCl). In all cases, the cooperative affinity is estimated to be in the range of 10^7^-10^8 ^M^-1^.

### Thermostability

The half-lives of the ssDNA-binding activities of *Tma*SSB and *Tne*SSB at 100°C, determined by gel mobility shift assays, were 10 h and 12 h, respectively. The thermostability for *Taq*SSB was 30 s at 95°C, 3 min at 90°C and 15 min at 85°C, as was also shown by Dąbrowski et al. [6].

**Figure 6:**
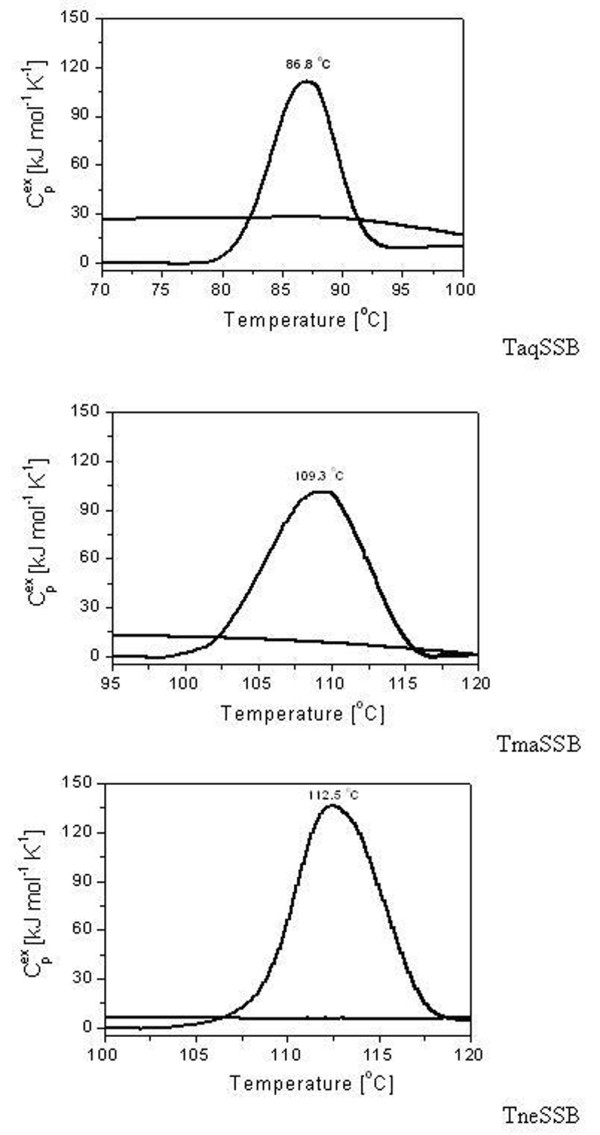
**DSC thermograms of SSB proteins**. Samples containing 1.5 mg/ml SSB were analyzed in 50 mM potassium phosphate buffer pH 7.5 and 0.1 M NaCl.

When analyzed by differential scanning microcalorimetry (DSC) the thermal unfolding of *Tma*SSB, *Tne*SSB and *Taq*SSB was found to be an irreversible process, as seen in the rescan thermograms (Figure 6). The *Tne*SSB had the highest thermostability, with a melting temperature (*T*_m_) of 112,5°C, whereas *Tma*SSB had a *T*m of 109,3°C (Figure 6). The melting temperature of *Taq*SSB was only 86,8°C. This difference in *T*_m _confirmed the different thermostabilities of the proteins indicated by the observed half-lives of the ssDNA binding activities. The thermograms of these SSB proteins did not show any characteristic signs of heavily aggregated proteins after heat denaturation. Moreover, the results of the DSC and the half-lives of the ssDNA binding activities suggest that the loss of binding activity of *Tma*SSB, *Tne*SSB and *Taq*SSB was connected with an irreversible thermal unfolding of the proteins.

In summary, the results showed that *Tma*SSB and *Tne*SSB are the most thermostable SSB proteins identified to date.

## Discussion

In this study, we have described the purification and characterization of SSB proteins from the thermophilic bacteria *T. maritima *and *T. neapolitana*. The results of the sequence analysis verified that a ssDNA binding domain (the first 106 amino acid residues) in one monomer of both *Tma*SSB and *Tne*SSB proteins possess a canonical oligonucleotide binding fold (OB-fold), very similar to the observed in the structure of *E. coli *SSB [23,24]. Both *Tma*SSB and *Tne*SSB form tetramers in solution as was shown by the gel filtration chromatography experiments. Furthermore, they possess the shortest and most acidic C-terminal domains yet identified (from 107 to 141 or 142 amino acid residues, respectively). The C-terminal domains contain 40% and 41.7% negatively charged amino acids, respectively.

Studies of other SSBs have often shown that the size of the binding site depends on the salt concentration. For example, for *Eco*SSB, at least two distinctly different DNA-binding modes have been described [3]. In high salt concentrations, 65 nt bind per *Eco*SSB tetramer with almost 90% fluorescence quench, whereas in low salt concentrations 35 nt are sufficient to saturate the protein and quench its fluorescence by only 53%. This phenomenon has also been demonstrated for all known *Deinococcus-Thermu*s SSBs [6,13-16]. However, such a distinctly different binding mode in high salt concentrations was not observed for the *Tma*SSB and *Tne*SSB proteins. The agarose gel mobility assays indicated that the binding site per tetramer is salt independent and is approximately 68 nucleotides based on fluorescence spectroscopy.

*Tma*SSB and *Tne*SSB proteins originating from the same genus, *Thermotoga*, showed quite similar thermostability (measured with an indirect method), i.e. 10 h and 12 h at 100°C, respectively. Both proteins possessed a higher thermostability than even the most thermostable *Tte*SSB2, which maintained full activity even after 6 h of incubation at 100°C [11]. Additionally, the results of differential scanning microcalorimetry (DSC) also demonstrated a very high thermostability of both the SSB proteins. *Tne*SSB had a higher thermostability (*T*_m _of 112,5°C) than *Tma*SSB (*T*m of 109,3°C), whereas in comparison the melting temperature of *Taq*SSB was only 86,8°C. Therefore the thermostability of *Tma*SSB or *Tne*SSB was much higher in comparison to the thermostability of homodimeric SSBs from the thermophilic *T. aquaticus, D. radiopugnans *[15] and *D. murrayi *[14]. In conclusion, the *Tma*SSB and *Tne*SSB are the most thermostable SSB protein identified up to date, offering an attractive alternative for *Taq*SSB and *Tth*SSB for applications in molecular biology and for analytical purposes especially for PCR and RT-PCR.

None of the two SSB proteins from *Thermotoga *seemed to possess any special features relative to *Eco*SSB and compared with other known thermostable SSBs. Neither their relative content of different amino acids nor the sequence comparisons could fully explain the cause of their exceptional thermostability. However, there were certain differences in the content of some amino acid residues. For example, the space between the highly hydrophobic core monomer and the highly acidic C-terminal fragment is very short in the *Tma*SSB and *Tne*SSB proteins in comparison with *Eco*SSB. This has also been demonstrated for SSBs from other highly thermophilic microorganisms like *T. aquaticus *and *T. thermophilus *[6]. This characteristically short and flexible C-terminus could protect the protein from thermal denaturation and make it more thermostable [6].

Based on the structure data the *Tma*SSB and *Eco*SSB proteins (without their flexible C-termini) [30,24] were analyzed to find more clues about the thermostability of SSBs from *Thermotoga*. The homology modeling of the protein regions which lack electron density was carried out using Modeller version 9.2 [31]. The modeled residues were 24 and 25, 38 to 48, 86 to 92 of *Tma*SSB and 1 and 2, 24 to 27, 40 to 49 of *Eco*SSB.

Thermostability seems to be a property acquired by a protein through a combination of many small structural modifications that are achieved with the exchange of some amino acid residues for others and the modulation of the canonical forces (e.g. hydrogen bonds, disulfide bonds, ion-pair interactions, hydrophobic interactions) found in all proteins [32]. The molecular mechanisms of thermostability are varied and depend on the specific protein [33]. The factors contributing to the protein stability include additional intermolecular interactions (e.g. hydrogen bonds, disulfide bonds, ion-pair interactions, hydrophobic interactions) and good general conformation structure (i.e. compact packing, more rigid, conformational strain release) [32].

**Figure 7:**
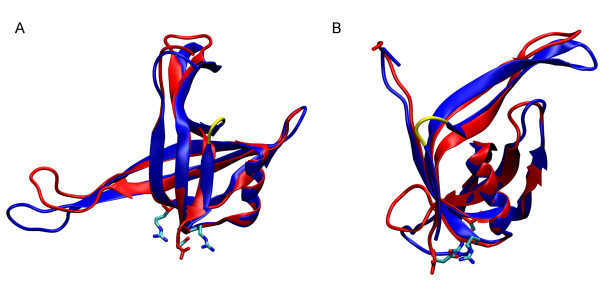
**Structural superposition of the DNA-binding domain of the *Tma*SSB and *Eco*SSB**. Two views of superposition of *Tma*SSB (red) and *Eco*SSB (blue) rotated against each others to visualized salt bridge and flexible loop. The superposition indicates a structurally conserved core with flexible loops. (A) The discussed salt bridge *Tma*SSB protein between Asp108 (red) and Arg12 (light blue) and Arg73 (light blue). (B) The additional flexible loop of *Eco*SSB (yellow). Structures prepared with using VMD version 1.8.7 [37].

The structural similarity between the *Tma*SSB and *Eco*SSB proteins is quite high but there are many characteristic features in the structures of *Tma*SSB monomer and tetramer which account for the thermostability [Tab. 1]. The amount of salt bridges in thermophile proteins is higher than in the equivalent proteins of mesophiles. The number of salt bridges in the tetramer of *Tma*SSB is by over 50% higher than in the *Eco*SSB tetramer, whereas in the *Tma*SSB monomer it is even by 100% higher than in the *Eco*SSB. A few of the *Tma*SSB salt bridges are particularly important for the protein stability, e.g. one of them which stabilizes the C-terminus (Figure 7A). It was showed that protein thermostability is correlated with the number of hydrogen bonds. The terminal β-strand (β6) of *Tma*SSB is a single long strand stabilized by the hydrogen bonds with the residues of the preceding antiparallel β-strand (β5), whereas in *Eco*SSB there are two shorter β-strands (β45_2 _and β5) divided by an additional loop that destabilizes this important region (Figure 7B). These two intermolecular interactions, stabilize this essential protein region thus enhancing the anchoring the *Tma*SSB C-terminus. The amino acid sequence alignments of thermophilic and the mesophilic proteins have displayed some significant substitutions in thermophilic proteins such as Gly to Pro [34]. The OB-fold of *Tma*SSB protein has a threefold higher content of Pro residues, whereas the content of Gly residues is twice lower than that of *Eco*SSB [Tab. 1]. Furthermore, there are three loops containing Pro residues in the *Tma*SSB protein and there is only one in *Eco*SSB, which makes the former less susceptible to unfolding than the latter.

**Table 1:** Results of structural comparison *Tma*SSB and *Eco*SSB proteins. Packing density calculated by means Voronoia software and procedure described in [38].

	Packing Density	Cavities	Amino acid residues		Shape correlation statistic (Sc)	
			
			Pro	Gly	(A/B)	(AB/CD)
Monomer *Eco*SSB	0.73	1	2	12	0.68	0.56

Tetramer *Eco*SSB	0.71	16	8	48		

Monomer *Tma*SSB	0.74	1	6	6	0.77	0.74

Tetramer *Tma*SSB	0.72	12	24	24		

Enhanced molecular compactness can enhance thermal stability. Compactness can be achieved by e.g. optimized packing or the elimination of unnecessary cavities [35]. The packing density of both a monomer and tetramer is slightly higher in *Tma*SSB whereas the number of cavities is as much as 25% higher in *Eco*SSB.

In order to examine the geometrical fit between the surfaces A and B subunits and AB and CD pairs of SSB proteins [30,24], the shape correlation statistic (Sc) [36] for *Tma*SSB and *Eco*SSB interfaces were calculated. This statistic provides a measure of packing of two protein surfaces. A value of Sc = 0 indicates no geometrical fit, whereas a value of Sc = 1 corresponds to two perfectly packed surfaces. Calculation of the shape correlation statistic gave a value of Sc = 0.68 or 0.77 for the interface of monomers A/B *Eco*SSB and *Tma*SSB, respectively. But surprisingly even more difference was for this parameter for interfaces between paired monomers AB/CD that equals 0.56 and 0.74 for *Eco*SSB and *Tma*SSB, respectively. These results indicate specifically that geometrical fit between *Tma*SSB protein surfaces is incomparably higher than *Eco*SSB.

In *E. coli*, the SSB base-stacking residues are Trp-40, Trp-54, Phe-60, and Trp-88, and in both *Tma*SSB and *Tne*SSB the related residues are Phe-31, Phe-52 or Phe-53, Phe-58 or Phe-64 and Trp-86 (Figure 1). Highly conserved His-55, Gln-76 and Gln-110, important for homotetramerization of *Eco*SSB, were not found in the SSB proteins from *Thermotoga*.

## Conclusions

We report here the purification and characterization of *T. maritima *and *T. neapolitana *SSBs, and how they relate to, and differ from, other members of this important class of proteins.

The *Tma*SSB and *Tne*SSB are the smallest known bacterial SSB proteins, their molecular mass deduced from the 141 and 142 amino acid sequences were 16.30 and 16.58 kDa, respectively.

The half-lives of *Tma*SSB and *Tne*SSB were extremely long: 10 h and 12 h at 100°C, respectively. When analyzed by differential scanning microcalorimetry (DSC) the melting temperature (*T*_m_) was 109.3°C and 112.5°C for *Tma*SSB and *Tne*SSB, respectively. These results were very surprising in the context of half-life of SSB proteins from thermophilic *Thermus *and *Deinococcus*.

The results showed that *Tma*SSB and *Tne*SSB are the most thermostable SSB proteins identified to date and those thermostability of both SSB proteins offer an attractive tool for many applications in molecular techniques, especially for thermal nucleic acids amplification methods (e. g. PCR).

## Methods

### Bacterial strains, plasmids, enzymes and reagents

*Thermotoga maritima *MSB8 (DSM 3106) and *T. neapolitana *(DSM 4359) were purchased from DSMZ (Deutsche Sammlung von Mikroorganismen und Zellkulturen GmbH, Germany). The *E. coli *TOP10F' (Invitrogen, USA) and BL21(DE3)pLysS (Novagen, UK) strains were used for genetic constructions and proteins expression, respectively. The reagents for PCR, the oligodeoxynucleotides, and the oligonucleotides 5'-end-labelled with fluorescein were purchased from DNA-Gdańsk II (Poland). Restriction enzymes, IPTG, and agarose were from Fermentas (Lithuania). The plasmid pET30Ek/LIC (Novagen, UK) was used for construction of the expression system. The reagents for protein purification were purchased from Sigma-Aldrich (USA).

### Cloning the ssb genes from T. maritima and T. neapolitana

Chromosomal DNA from *T. maritima *and *T. neapolitana *was isolated using the Genomic DNA AX Bacteria kit (A&A Biotechnology, Poland). In the *T. maritima *(GenBank accession no. AE000512) genome, the *ssb *gene is flanked by the conservative *rpsF *and *rpsR *genes encoding the ribosomal proteins S6 and S18. Hence, primers complementary to the most conservative regions of those genes were designed and synthesized for PCR amplification. The forward primer was 5'-GGGTATGAGAAAGTTCGCCT (20 nt) and the reverse primer was 5' ATCTGTCTTGCCCTTTTGATG (21 nt). PCR reactions were performed using 1U of *Pwo *polymerase (DNA-Gdańsk II, Poland) in 50 μl buffer containing 10 mM KCl, 20 mM Tris-HCl pH 8.8, 10 mM (NH)_2_SO_4_, 0.1% Triton X-100, 2 mM MgSO_4_, 1 mM dNTPs, 0.4 μM of each primer and approximately 200 ng of *T. maritima *or *T. neapolitana *DNA. Forty cycles were performed with a temperature profile of 60 s at 94°C, 90 s at 54°C and 120 s at 72°C. Specific PCR products, about 900 bp, were obtained and sequenced to confirm the presence of *ssb*-like gene.

Based on the *ssb *gene sequences from *T. maritima *and *T. neapolitana*, gene-specific primers for PCR were designed and synthesized. PCR was carried out using the forward 5'-GCGCAT***ATG*TCTTTCTTCAACAAGATC **(27 nt) and reverse 5'-ATAAGCTTAA***TCA*AAATG GTGGTTCATC **(28 nt) primers for the *ssb *gene of *T. maritima *and the forward 5'- GCGCAT***ATG*TCTTTTTTCAACAGGATC **(27 nt) and reverse 5'- ATAAGCTTAA***TCA*GAATGGCG GTTCGTC **(28 nt) primers for the *ssb *gene of *T. neapolitana*. The boldface parts of the primer sequences are complementary to the nucleotide sequences of the *ssb *genes in *T. maritima *and *T. neapolitana*, respectively, whereas the 5' overhanging ends of the primers contain recognition sites for restriction endonucleases and are designed to facilitate cloning (the *Nde*I and *Hin*dIII recognition sites are underlined; the ATG start codon and TGA stop codon are shown in italics). The PCR conditions were the same as described above. Both PCR products (0.5 μg) were digested with *Nde*I and *Hin*dIII and analyzed by electrophoresis on a 1% agarose gel stained with ethidium bromide. Specifically, approximately 420 bp amplification products were cut out of the gel and purified using the Gel-Out AX kit (A&A Biotechnology, Poland). The purified DNA fragments were ligated into pET30Ek/LIC between the *Nde*I and *Hin*dIII sites. *E. coli *strains TOP10F' cells were transformed with the ligation mixtures and the colonies obtained were examined for the presence of *ssb *genes from *T. maritima *and *T. neapolitana *by PCR amplification and restriction analysis. Single clones, named pETSSBTma and pETSSBTne, were selected and sequenced to ascertain the authenticity of the clones. The constructed plasmids were used in the expression and purification procedure described below.

### Protein sequence analysis of the TmaSSB and TneSSB

The amino acid sequences of the *Tma*SSB and *Tne*SSB proteins were analyzed using standard protein-protein BLAST and RPS-BLAST. Multiple sequence alignments were created using the program MAFFT and the results were analyzed and edited using the editor program GeneDoc (copyright by Karl Nicholas). Dendogram of the amino acid sequences of SSB proteins were edited using the editor program Dendroscope [25].

### Expression and purification of the TmaSSB and TneSSB

The *E. coli *BL21(DE3)pLysS strain transformed with pETSSBTma or pETSSBTne was grown at 37°C in 0.5 L LB containing 34 μg/ml kanamycin and 50 μg/ml chloramphenicol to an OD_600 _of 0.4. Expression was then induced by addition of IPTG to a final concentration of 0.5 mM. After 6 h, the cells were harvested by centrifugation, and suspended in 50 ml buffer A (20 mM Tris-HCl pH 8.0, 50 mM NaCl, 1 mM EDTA, 0.1% Triton X-100). The purification procedure was very similar to the previously published purification scheme for the SSB from calf thymus [26], and that for thermostable SSB proteins [6]. Generally, the cells were disrupted by sonication and the insoluble debris were removed by centrifugation. The supernatant was heat-treated at 80°C for 20 min and denatured mesophilic proteins were discarded by centrifugation. This supernatant was directly loaded on a QAE-cellulose column (50 ml bed volume, Sigma-Aldrich, USA), from which the proteins were eluted with a linear gradient of 0.05-2 M NaCl in buffer B (20 mM Tris-HCl pH 8.0, 50 mM NaCl, 1 mM EDTA). The SSB-containing fractions, detected by SDS-PAGE, were combined and loaded on a ssDNA-cellulose column (5 ml, USB, USA). SSB proteins were eluted with gradient of 0.5-1.5 M NaCl and 50% ethylene glycol. The fractions with SSB proteins were collected and dialyzed against buffer B, concentrated using an Amicon Ultra-10 centrifugal filter device (Millipore, USA), and stored at -20°C in buffer C (20 mM Tris-HCl pH 8.0, 50 mM NaCl, 1 mM EDTA, 50% glycerol, 0.05% Igepal) until used. The purity of *Tma*SSB and *Tne*SSB proteins was examined by the optical densitometry on the SDS-PAGE gel and the amounts were estimated spectrophotometrically using the appropriate absorption coefficient factor.

### Estimation of the native molecular mass

The molecular mass of the *Tma*SSB and the *Tne*SSB protein was determined by two independent methods: (i) FPLC gel filtration on a Superdex HR 75 column (Amersham Bioscience AB, Sweden), (ii) optimized chemical cross-linking experiments using 0.1% (v/v) glutaraldehyde for 1-30 min with *Tma*SSB or *Tne*SSB concentrations between 50 and 500 μg/ml [27]. Bovine albumin (66 kDa), ovalbumin (43 kDa), carbon anhydrase (29 kDa) and cytochrome C (12.4 kDa) were used as standard proteins for calibration in the gel filtration assay.

### Gel mobility shift assays: binding to ss oligonucleotides

A fixed quantity (10 pmol) of 5'-end fluorescein-labelled oligonucleotides (dT)_35_, (dT)_60_, (dT)_76 _or (dT)_120 _or ssDNA of phage M13 (1.5 pmol) was incubated for 20 min at 25°C with 10, 100 or 200 pmol of *Tma*SSB or *Tne*SSB in 10 μl of binding buffer (20 mM Tris-HCl pH 7.5, 1 mM EDTA) containing 2 mM or 100 mM NaCl. Next, the reaction products were loaded onto 2% agarose gels without ethidium bromide and separated by electrophoresis in TAE buffer (40 mM Tris acetate pH 7.5, 1 mM EDTA). The bands corresponding to the unbound ssDNA, and the various SSB-ssDNA complexes following ethidium bromide staining were visualized by UV light and photographed.

### Fluorescence titration

Fluorescence was measured with a Perkin-Elmer LS-5B luminescence spectrometer as described earlier [28]. For the binding reaction, 2 ml binding buffer (20 mM Tris-HCl pH 7.5, 1 mM EDTA) containing 2 or 100 mM NaCl was used. A constant amount of *Tma*SSB or *Tne*SSB (1 nM) protein was incubated in the buffer at 25°C with varying quantities of (dT)_76 _oligonucleotide (from 0 to 0.8 nM). The excitation and emission wavelengths were 295 and 348 nm, respectively. The binding curve was analyzed using the model as described by Schwarz and Watanabe [29] with *n *as binding site size, *ω·K *as cooperative binding affinity and fluorescence quench *Q*_f _as parameters. Fluorescence quench is defined as 1 -F_bound_/F_free_, where F_free _and F_bound _denote the fluorescence intensities measured for free and nucleic acid bound protein, respectively

### Thermostability

To determine the thermostability of the *Tma*SSB and *Tne*SSB proteins, both an indirect and a direct (differential scanning calorimetry, DSC) method was used.

In the indirect method, a fixed quantity (10 pmol) of a 5'-end fluorescein-labeled oligonucleotide (dT)_35 _was added to 10 pmol of *Tma*SSB, *Tne*SSB or *Taq*SSB (control sample) preincubated at 85 °C, 90 °C, 95 °C and 100 °C for 0, 1, 3, 5, 10, 15, 30, and 60 min in 10 μl binding buffer containing 100 mM NaCl. In further experiments with the *Tma*SSB and *Tne*SSB proteins, the incubation times at 100°C were increased to 2, 4, 8, 10, 11 and 12 h. After 20 min incubation at 25 °C, the protein-DNA complexes were separated from free DNA by electrophoresis on a 2% agarose gel, and 50% quantities of protein-(dT)_35 _complex were evaluated by densitometric analysis using the VersaDoc imaging system and the QuantityOne software (BioRad, USA).

Microcalorimetric measurements were performed using a NanoDSC microcalorimeter (Calorimetry Science Corporation, USA). Samples containing 1.5 mg/ml SSB in 50 mM potassium phosphate buffer pH 7.5 and 0.1 M NaCl were analyzed. The calorimetric scans were carried out between 20 and 130°C with a scan rate of 1°C/min (Figure 6). The reversibility of the transition was checked by cooling and reheating the same sample with the scan rate of 1°C/min. Results from the DSC measurements were analyzed with the NanoAnalyze Software V 1.1 (TA Instruments, USA).

### Nucleotide sequence accession number

The nucleotide sequences of the *ssb *genes of *T. maritima *and *T. neapolitana *are available in the GenBank database under the accession numbers AAD35689[20] and GU125728, respectively.

## List of abbreviations used

dsDNA: Double-stranded DNA; OB fold: Oligonucleotide/oligosaccharide-binding fold; RPA: Replication protein A; SSB: Single-stranded-DNA-binding; ssDNA	: Single-stranded DNA.

## Authors' contributions

MO conceived of the study, carried out the molecular genetic studies, participated in the design of the study and drafted the manuscript. AG, MN and MM carried out the molecular genetic studies. MW performed homology modeling of *Tma*SSB and *Eco*SSB. JK participated in design of study and drafted the manuscript. All authors read and approved the final manuscript.
